# Atlas Fracture with Concomitant Vertebral Artery Hypoplasia, a Rare but Potentially Hazardous Combination: A Case Report

**DOI:** 10.7759/cureus.4172

**Published:** 2019-03-04

**Authors:** Georgios Vynichakis, Theodoros B Grivas, Hippocrates Moschouris, Dimitrios Filippou, Panagiotis Skandalakis

**Affiliations:** 1 Orthopaedics, General Hospital of Piraeus Tzaneio, Piraeus, GRC; 2 Orthopaedics and Traumatology, General Hospital of Piraeus Tzaneio, Piraeus, GRC; 3 Radiology, General Hospital of Piraeus Tzaneio, Athens, GRC; 4 Surgery, Medical School of National and Kapodistrian University of Athens, Athens, GRC

**Keywords:** cervical spine, c1 fracture, atlas fracture, jefferson fracture, vertebral artery, vertebral artery hypoplasia, vertebral artery injury, cervical spine trauma, posterior circulation ischemia

## Abstract

The fractures of the first cervical spine vertebrae (atlas) represent 7% of all the overall cervical spine fractures. Hypoplasia of the vertebral artery is also rare (10% of the general population), but even rarer is the combination of those both conditions. This combination should always be identified and treated because sometimes it can be extremely dangerous for the patient.

We present a case of a 24-year-old patient who suffered an atlas fracture with concomitant vertebral artery hypoplasia (VAH). We also present the diagnostic algorithm and the treatment management that we have followed.

In case of cervical spine trauma the neurovascular symptoms should not be underestimated. Any neurological symptom (sensory, motor, reflex deficits) should be evaluated in detail. In some cases, with uncommon neurological symptoms such as, in our case, unilateral headache, dizziness and vertigo (or generally, involuntary eye movements and salivation, impaired speech and hearing, diplopia, blur vision, incoordination, imbalance, limb weakness) head injury or vertebral artery (VA) injuries have to be suspected. Further evaluation with brain computed tomography (CT) scan and computed tomography angiography (CTA) should be provided. In case of cervical spine trauma over a pre-existing VAH the complications rate is even higher and the early diagnosis and treatment are crucial.

## Introduction

The fractures of the first cervical vertebra (atlas) amount to almost 7% of all cervical (C) spine fractures [1]. The risk of neurological injury is low and, usually, these fractures are not diagnosed due to inadequate imaging of the occipitocervical junction in plain radiographs. History of head injury, especially on the top, and upper neck pain are suspicious for atlas fracture. Common causes of injury are car accidents, motor accidents and head trauma during diving. Almost 50% of atlas fractures are associated with one more concomitant spine injury such as second cervical vertebra (axis) fractures (40% of the cases). The most common classification of atlas fractures is the Landells and Van Peteghem classification [2-5].

Vertebral artery hypoplasia (VAH) was first described in the 19th century. Generally, there is an ongoing debate concerning the definition of VAH. VAH ranges from less than 2 mm diameter to less than 3 mm or an asymmetry ratio of equal or greater than 1:1,7 [6]. Many authors suggest that VAH is more frequent on the right side. Left vertebral artery (VA) is dominant in 70% of individuals [7-8]. The VAs are major arteries of the neck supplying a component of the vertebrobasilar vascular system (upper spinal cord, brainstem, cerebellum, posterior part of the brain). VAs origin from subclavian arteries and ascend cranially on either sides of the spinal column [9-10]. Due to their location, VA injuries are potential on cervical spine (CS) trauma. VA injuries make up less than 5% of all cervical arteries injuries. Almost 75% of VA injuries are asymptomatic because of its location and because the contralateral VA will most likely (85%) provide sufficient flow to the posterior circulation [10].

The brain is the most energy-intensive organ and one of the most perfused organs of the human body. It uses nearly 50% of the human body's glucose, although it is 2% of total body mass. The brain perfusion provided by the anterior circulation (originates in the internal carotid arteries) and the posterior or vertebrobasilar circulation (originates in the vertebral arteries) [9]. Clinical features when the blood supplies decrease are: headache, brainstem dysfunction, risk of ischemic events (such as stroke), repeated episodes of hemiparesis (associated with bi-temporal throbbing headache and vomiting) and cerebellar disorders [6, 8].

The VAH is a rare anatomical variant but it is also well described in the literature that VAH is a predisposing factor for posterior brain ischemia and brain strokes. Given the fact that there is an association of VA injury with cervical spine trauma, the combination of cervical spine trauma with a VAH and VA injury could be hazardous for the brain blood flow and has to be early identified by the physician. Except from the clinical examination, there are appropriate tests to diagnose VAH and VA injury such as ultrasonography, digital angiography (DA) or digital subtractive angiography (DSA), computed tomography angiography (CTA) and magnetic resonance angiography (MRA) [11].

## Case presentation

**Figure 1:**
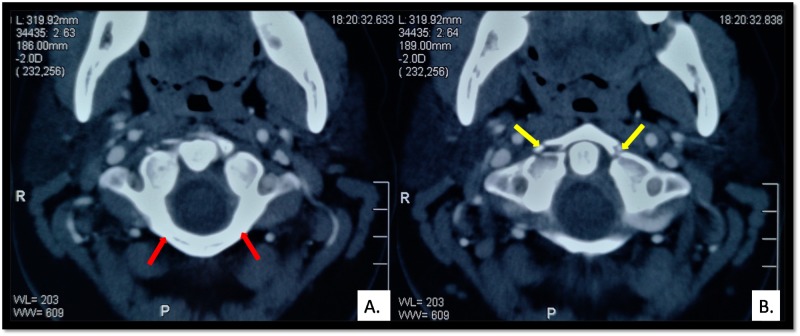
Jefferson-type II fracture with normal ADI: (A) Intact posterior arch of atlas (red arrows). (B) Bilateral fracture of the anterior arch of atlas (yellow arrows). ADI: Atlantodens Interval

**Figure 2:**
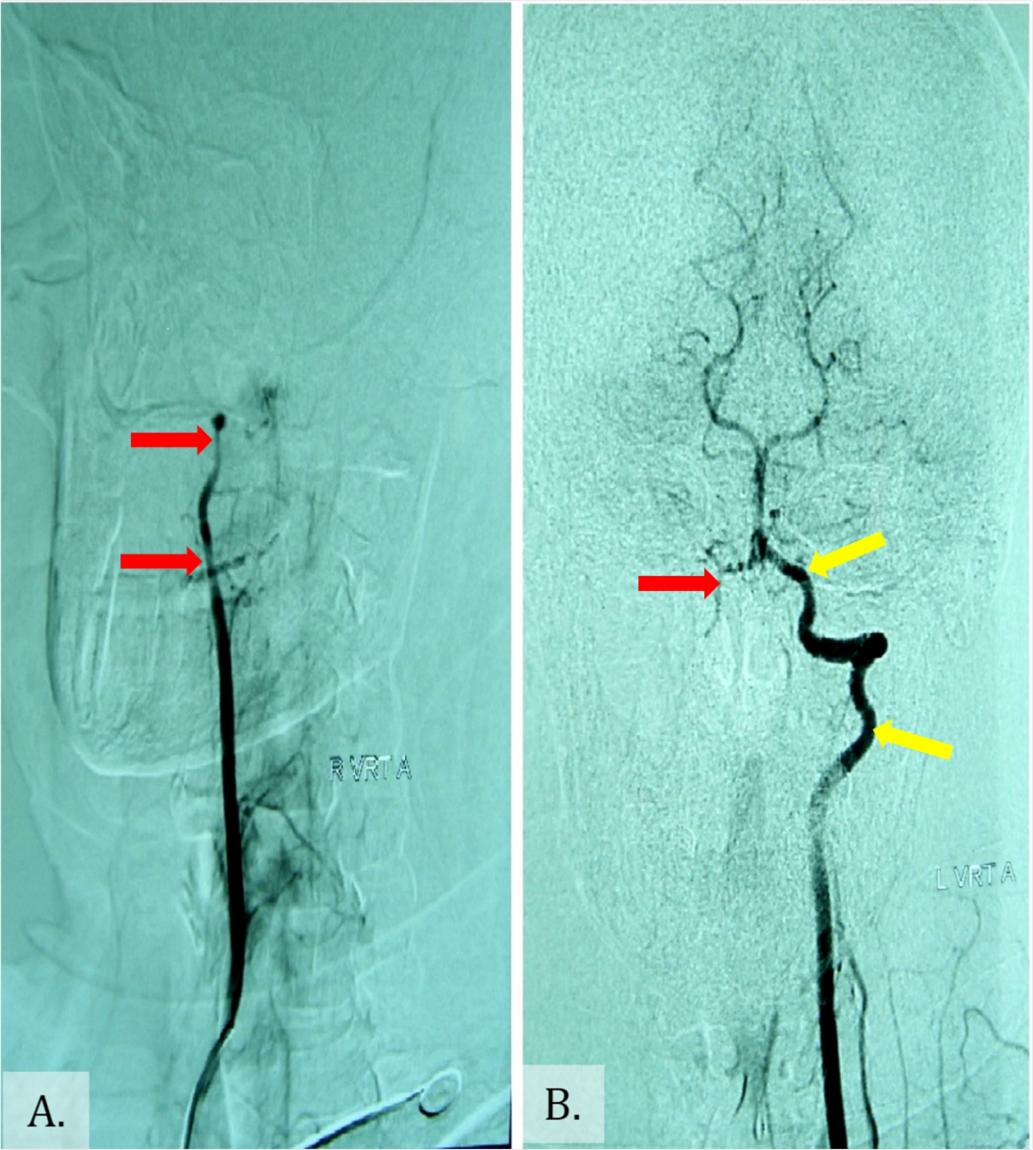
DSA: (A) Right VA and (B) left VA. The right VA seems to be narrower (red arrows) than the contralateral left VA (yellow arrows) which appears normal. DSA: Digital Subtractive Angiography; VA: Vertebral Artery

**Figure 3:**
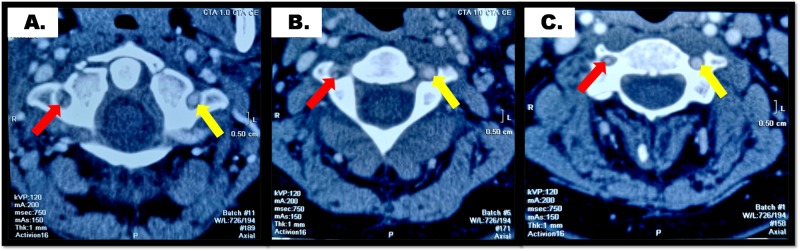
CTA scan: Hypoplastic right VA (red arrows) and normal left VA (yellow arrows) at (A) atlas level, (B) axis level and (C) third cervical vertebrae level. CTA: Computed Tomography Angiography; VA: Vertebral Artery

**Figure 4:**
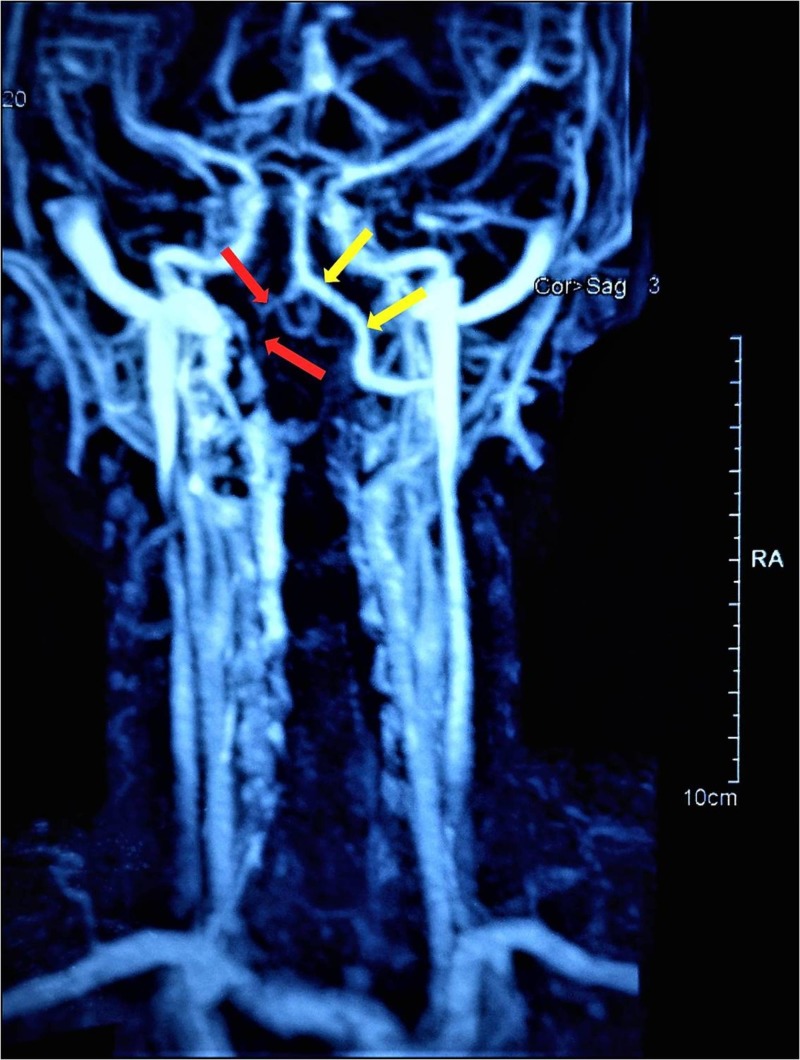
MRA: Hypoplastic right vertebral artery (red arrows). The right VA (red arrows) seems to be narrower than the contralateral left VA (yellow arrows). MRA: Magnetic Resonance Angiography; VA: Vertebral Artery

A 24-year-old female presented non-ambulated to the emergency department of our hospital after a car accident. She was well orientated (Glasgow Coma Scale: 15/15) and was complaining of head and neck pain, dizziness, vertigo and vomiting. No other injuries except a superficial wound of the head were registered. Regarding the personal medical history, occasional headaches and vertigo treated with conservative treatment were referred from the patient. After plain radiographs and whole body computed tomography (CT) scan (our hospital's trauma protocol), no brain damage and an atlas fracture (Jefferson-type ΙΙ) with normal atlantodens interval (ADI) was identified (Figure 1). As a stable fracture, the treatment was decided to be conservative and the head was immobilized into a hard cervical collar. Furthermore, the patient remained hospitalized for clinical monitoring. Forty-eight hours later, the patient continued to complain of unilateral headache and dizziness. A further evaluation with DA, CTA and brain CT scan was decided, in order the brain blood inflow and the brain parenchyma to be evaluated. No abnormal findings were detected from the brain parenchyma but the presence of unilateral VAΗ was identified (Figures 2, 3) and further evaluation with MRA was performed for confirmation (Figure 4).

After vascular surgeon’s consultation, the patient started subcutaneous low molecular weight heparin (LMWH) and remained hospitalized until the symptoms improved. She continued taking LMWH for three weeks totally.

She had a follow-up at 10 and 20 days and at two and three months post-injury. After two months, when the fracture was healed and the hard collar was removed, she started to mobilize slightly. At the two-month follow-up, no neurological symptoms were registered and after three months she had totally recovered. At three weeks post-injury, after vascular surgeon’s consultation, the patient’s treatment for the VAH changed from LMWH to antiplatelets.

## Discussion

**Figure 5:**
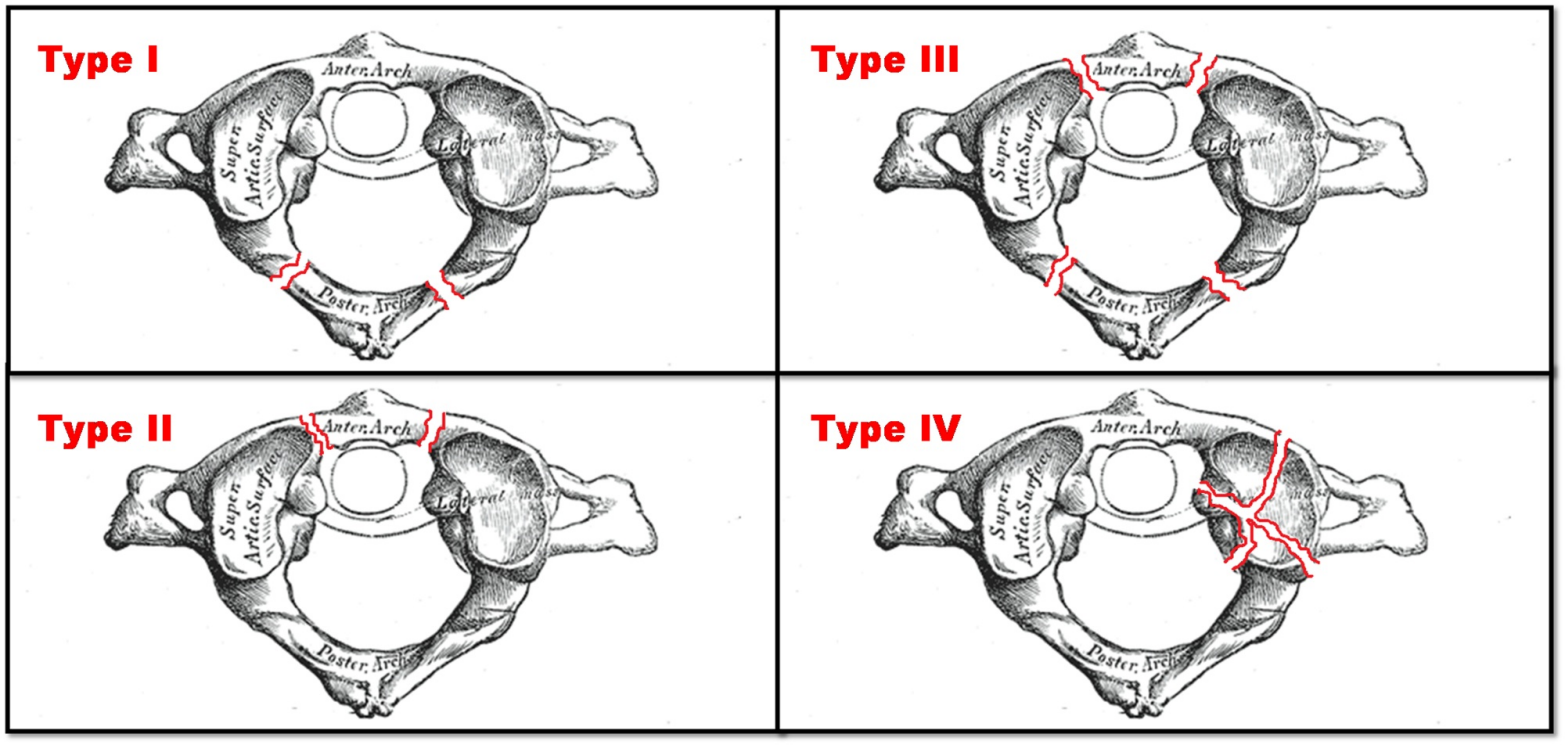
Jefferson classification of atlas fractures: Type I: Fracture of the posterior arch only, Type II: Fracture of the anterior arch only, Type III: Fracture of both the anterior and posterior arches, Type IV: Fracture of the lateral mass(es) of atlas with or without disruption of the transverse atlantal ligament. Figure is modified from original in Gray's Anatomy of Human Body (1918), licensed under Public Domain. Conflict of interest: none

Atlas fractures account 2-13% of all CS injuries and 1-2% of all spine injuries [1-2]. Sir Geoffrey Jefferson was the first to report fractures in the posterior and anterior arch of atlas in 1920 (Figure 5) [2-3]. Landell recently modified the Jefferson Classification, and this modification is used by the majority of the surgeons (Table 1). The difficulty in diagnosis is mainly due to pain absence and obscure neurological signs. Patients with atlas fractures present with pain in the upper neck and often refer history of head trauma. Usually, they do not present with neurological deficit.

**Table 1:** Landell and Van Peteghem Classification.

Type	Pathology	Stability	Treatment	Consider to Jefferson’s Classification
I	Fracture of either the anterior or posterior arches of atlas (but not both)	Stable	Conservative: hard collar	Type I (posterior arch) or Type II (anterior arch)
II	Fractures of both anterior and posterior arches, is called a "burst" fracture (also known as a Jefferson's fracture)	Stability determined by integrity of transverse ligament	If intact, hard collar. If disrupted, Halo vest (for bony avulsion) or atlas-axis fusion (for intrasubstance tear)	Type III
III	Unilateral lateral mass fracture of atlas	Stability determined by integrity of transverse ligament	If stable, treat with hard collar. If unstable, Halo vest	Type IV

In any case, the history of the patient, a detailed clinical examination and radiographs are necessary for the diagnosis. The mechanism of injury and the symptoms of the patient should be evaluated. It is very important to be highlighted that, very often, additional radiographs, such as the mouth-open odontoid view, are necessary for the diagnosis, because no abnormal findings can be presented to the conventional anteroposterior (AP) and lateral radiographs. The evaluation of the symmetry of the axis is the most important fact on the odontoid view and additionally, the ADI is the most useful measurement on the lateral view of the cervical spine. ADI > 3 mm (5 mm for children) is suspicious for atlas fracture [3, 5]. Further evaluation with CT scan and magnetic resonance imaging (MRI) scan is necessary for the classification of the fracture and the identification of any ligamentous or spinal cord injury. The treatment of the stable atlas fractures is conservative for types I and II and surgical for unstable type II and III fractures (Table 1). The conservative treatment includes hard cervical collar or Halo (for six to 12 weeks) and the surgical treatment is usually the atlas-axis fusion with or without occipitocervical fusion [3-5]. Surgical treatment preferred in nonunion or malunion (when symptoms presented).

Cases of CS fractures are more complex to manage if they combine with another pathology such as VAH or VA injury. VAH was first described in the 19th century. Generally, there is an ongoing debate concerning the definition of VAH. VAH ranges from less than 2 mm diameter to less than 3 mm or an asymmetry ratio of equal or greater than 1:1,7 [6]. The VAs are major arteries of the neck, supplying component of the vertebrobasilar vascular system and the circle of Willis (upper spinal cord, brainstem, cerebellum, posterior part of the brain) [8-9]. Left vertebral artery is dominant in 70% of individuals and up to 10% may have unilateral hypoplasia [7-8]. We understand the importance of VAs but very little is known about their clinical relevance [6]. There are several studies in the literature which describe the clinical significance of VAH in case that the posterior blood flow to the brain may be decreased. Almost 20 years ago, VAH found to be a predisposing factor for posterior brain ischemia and brain strokes [12-13]. In other words, is VAH relevant with migraine, vestibular evoked myogenetic potential (VEMP), vestibular neuronitis? The most important question is if VAH is a risk factor for posterior circulation stroke (PCS). Several researches tried to answer this question and a lot of risk factors for circulation stroke such as diabetes, smoking, hyperlipidemia and hypertension have been described and proposed. VAH is considered as another one risk factor for PCS [6, 14]. A hypoplastic VA presents significantly lower flow volume and decreased flow velocities and seems to be more susceptible to pro-thrombotic or atherosclerotic processes than normal VA. The VA thrombi can cause in situ stoke and are prone to cause distal embolization [14]. As a result, it may cause various temporary or permanent clinical signs to the patients (such as headaches, dizziness, brainstem dysfunction, breathing problems, vertigo and nystagmus, involuntary eye movements and salivation, diplopia or blurred vision, impaired speech and hearing, incoordination, imbalance, ataxia, limb weakness) [8]. The therapy of VAH in most cases is based in anticoagulants administrated subcutaneously.

It is also well known that blunt cervical spine trauma can be associated with vertebral arteries injury (VAI). More often, a VAI may be silent (75% asymptomatic), especially when the non-dominant VA is injured, because the collateral blood flow from the intact contralateral VA (most likely 85%) or the anterior flow from the circle of Willis remains sufficient [7, 10-11]. However, a VAI can cause central migration of blood clots and crucial brain ischemia with various outcomes [11]. Clinical symptoms related to posterior brain ischemia include unilateral headache, vertigo and nystagmus, diplopia or blurred vision, dysarthria and dysphagia and altered consciousness. In rare cases, where bilateral or dominant VA injured, fatal consequences are probable (including reduction of consciousness level and sudden respiratory arrest) and increase morbidity and mortality [7]. Low grade and asymptomatic VA injuries could be treated by observation and anticoagulation therapy. High grade and symptomatic VA injuries are best treated by endovascular approaches or thrombolytic therapy [11]. There are no standard guidelines for the management of these complex pathologies. Follow-up with CTA at two weekly intervals is recommended to assess response to therapeutic intervention [7,15].

In our case, the patient with atlas fracture presented neurological symptoms (unilateral headache, dizziness, vertigo) which could not be associated with the fracture because the range of the spinal canal at this level is quite large so the spinal cord not to be easily compressed. As a result, symptoms which indicate brain dysfunction, without brain parenchyma damage, should alert the physicians for a suspicious VA disruption (the most characteristic clinical finding of VA injury is the unilateral headache) and further evaluation with CTA should be performed [5]. For our patient, with VAH, the possibility of complications after cervical spine trauma seems to be higher [11]. It is well reported in the literature that a hypoplastic VA seems to be more susceptible to pro-thrombotic or atherosclerotic processes than normal VA and therefore a possible VA injury in a pre-existing contralateral hypoplastic VA could be extremely hazardous for brain ischemia, temporary or permanent neurological deficits and death [16]. Under no doubt, the early radiological investigation and diagnosis of this condition, allowed us to treat the patient in an appropriate way (with LMWH for three weeks and continued with anti-platelets) [15]. Even though in minor symptomatology, as in our case, antiplatelet or anticoagulation therapy should be given.

In general, the treatment of VA injuries or VA flow deficits in combination with cervical spine trauma includes a wide range of procedures from acute thrombolysis, anticoagulation or antiplatelet therapy and in some cases endovascular or open repair-reconstruction of the VA [15].

On CS trauma the neurovascular symptoms should not be underestimated. The cooperation among orthopaedics, neurosurgeons and vascular surgeons is necessary, especially when the diagnosis or the symptoms are obscure, in order to ensure the appropriate treatment.

## Conclusions

Any cervical spine injury with uncommon neurological symptoms from the brain should raise suspicion of vertebral artery injury. Further evaluation has to be performed. In case of a pre-existing VAH, the complications rate is even higher and the early diagnosis and treatment are crucial. The correct diagnosis will ensure that the appropriate treatment should be applied either it is conservative or operative according to the protocols previously referred.
